# Assessing the impact of childhood pneumococcal vaccination on pneumonia mortality in Colombia: a 14-year analysis

**DOI:** 10.1186/s12889-025-23631-1

**Published:** 2025-09-01

**Authors:** C. I. Parellada, L. F. Reyes, J. Urrego-Reyes, J. L. Webster, P. C. Pungartnik, A C Dos Santos, M. Rojas, F. de la Hoz

**Affiliations:** 1 Outcomes Research Regional Latin America, MSD Brazil, São Paulo, SP Brazil; 2 Unisabana Center for Translational Science, School of Medicine, Universidad de la Sabana, Chía, Colombia; 3 Clínica Universidad de La Sabana, Chía, Colombia; 4 Outcomes Research Regional Latin America, MSD Colombia, Bogotá, Colombia; 5 Outcomes Research Regional Latin America, Merck & Co., Inc, Rahway, NJ USA; 6 Real-World Insights, IQVIA, São Paulo, SP Brazil; 7 Universidad Nacional de Colombia, Bogotá, Colombia; 8 Avenida Chucri Zaidan no. 296 São Paulo, São Paulo, 04583-110 Brazil

**Keywords:** Pneumococcal conjugate vaccines, Mortality, Pneumonia, *Streptococcus pneumoniae*

## Abstract

**Background:**

The 10-valent pneumococcal conjugate vaccine (PCV10) has been offered to all infants through Colombia’s National Immunization Program (NIP) since 2012, with catch-up vaccination until age 5. However, pneumococcal vaccination is not currently included in the NIP for other age groups, such as those ≥ 5 years with medical conditions or older adults. This study assessed the pediatric PCV10 effect on pneumonia mortality rate (MR) trends across different age groups from 2006 to 2019.

**Methods:**

This retrospective time-series study utilized the national death registration data. Deaths caused by pneumonia as the underlying cause of death were identified via ICD-10 codes and stratified by age groups (< 1, 1–4, 5–17, 18–49, 50–59, and ≥ 60 years). Crude MR, age-adjusted MR, and age-specific MRs per 100,000 population were calculated. Trends were assessed using joinpoint regression and expressed as annual percentage change (APC) and average APC in the pre-PCV10 (2006–2011), post-PCV10 (2013–2019), early post-PCV10 (2013–2016), and late post-PCV10 (2017–2019) periods.

**Results:**

From 2006 to 2019, there were 102,082 pneumonia-related deaths. The age-specific MR for infants < 1 year significantly decreased from 83.8 to 28.6 per 100,000 between 2006 and 2019 (APC:-6.9), while for children aged 1–4 years, it decreased from 9.3 to 3.8 (APC:-5.1). Other older age groups exhibited stable trends in the post-PCV10 period, except for adults aged ≥ 60 years, who had the highest age-specific MR (~ 112 per 100,000) over the study period, with an AAPC of 6.5% in the early post-PCV10 period, followed by stable trends in the late post-PCV10 period. The age-adjusted MR showed an increasing trend with an APC of 1.9% from 2008 to 2019.

**Conclusions:**

Our study found decreasing age-specific MR trends in children under 5 in the post-PCV10 period; however, no evidence of indirect benefits was seen in unvaccinated age groups older than 5 years. The findings underscore the need to expand pneumococcal vaccination programs to other age groups, mainly adults ≥ 60 years.

**Supplementary Information:**

The online version contains supplementary material available at 10.1186/s12889-025-23631-1.

## Introduction

Pneumonia remains a significant global health challenge, mainly due to its high mortality rate [1]. Between 1990 and 2019, it has been estimated that this illness was responsible for an annual average of 2.5 million deaths across all age groups [2]. Among bacterial pathogens, *Streptococcus pneumoniae* (*S. pneumoniae*) is the leading cause of pneumonia-related deaths, resulting in more deaths than all other etiologies combined [3]. Notably, this pathogen alone accounts for over 1 million deaths annually among the most vulnerable populations: young children and older adults [3].

In response to this significant burden, pneumococcal conjugate vaccines (PCVs) have been developed and introduced in many countries [4]. Following the implementation of PCV in infant immunization programs worldwide, a substantial reduction in the burden of pneumonia has been reported among children [4–8]. Additionally, evidence from various countries, such as the United States, United Kingdom, Canada, and South Africa, indicates that infant immunization protects unvaccinated age groups through herd immunity [9–12]. This is achieved by reducing the vaccine-serotype nasopharyngeal carriage in young children and its circulation in the community [13].

In Latin America, an estimated 143,947 people of all ages died from pneumococcal disease each year between 1990 and 2016, even in the post-pneumococcal vaccine era, with the majority of the burden being represented by pneumonia. Most studies in this region have predominantly focused on investigating the direct impact of pneumococcal vaccination on pneumonia mortality trends in children under five years [7, 14–16]. Research on the indirect impact of pediatric PCV programs on pneumonia mortality in unvaccinated older populations in Latin America is limited. A recent study conducted across five Latin American countries found no detectable effect in reducing pneumonia mortality in individuals ≥ 5 years [17].

Colombia, a middle-income country in Latin America, first introduced the PCV7 into the national immunization program in 2006, exclusively targeting infants at high risk or those with certain medical conditions [18, 19]. By 2012, the program was expanded to include all infants and transitioned to PCV10 using a 2 + 1 schedule at 2, 4, and 12 months of age. In 2022, PCV10 was replaced with PCV13 [18]. Sustained vaccination coverage rates for PCV have exceeded 80% for the final PCV dose since 2012 [19]. However, the universal pneumococcal vaccination program is not included in the NIP for individuals over 5 years with medical conditions or for older adults [20].

Therefore, this study aimed to assess the childhood PCV10 impact on pneumonia mortality rate trends across different age groups in Colombia from 2006 to 2019, including seven-year data post-PCV10 introduction. The findings from this study can contribute to a more comprehensive understanding of the population-level effect of the pediatric PCV program in unvaccinated older age groups. These insights can inform resource allocation, program prioritization, and strategies to reduce pneumonia mortality in the country.

## Materials and methods

### Study design and data sources

This ecological time-series study utilized secondary data from local databases in Colombia. To assess mortality due to pneumonia as the underlying cause of death, the number of deaths was extracted from the National Statistics Department (Departamento Administrativo Nacional de Estadística, DANE - Estadísticas vitals, EEVV) from 2006 to 2019, available at https://microdatos.dane.gov.co, using the International Classification of Diseases, Tenth Revision (ICD-10) [21]. Population estimates were obtained from DANE based on the 2018 census. Data on PCV10 vaccination coverage were extracted from the World Health Organization (WHO) and United Nations Children’s Fund (UNICEF) estimates of national immunization coverage (WUENIC), available at https://immunizationdata.who.int/, considering infants fully vaccinated with the 3-dose schedule [19]. All data from Colombian databases used in this study were anonymized and comply with Colombian Resolution 1409 of 2022, which permits the secondary use of anonymized data without ethics committee approval for scientific purposes or public policy formulation.

### Study population and case definitions

Pneumonia-related deaths were identified by using the following ICD-10 codes: J13 (pneumococcal pneumonia); J15, J15.0-9 (bacterial pneumonia, unspecified); J16, J16.0, J16.8 (pneumonia due to other infectious organisms, not elsewhere classified); J17, J17.0-3, J17.8 (pneumonia in diseases classified elsewhere) and J18.0, J18.1, J18.9 (pneumonia or bronchopneumonia, organism unspecified) (Table S1). The overall study population included individuals of all ages and was stratified into the following groups: < 1, 1–4, 5–17, 18–49, 50–59, and ≥ 60 years.

### Data analysis

The annual crude mortality rates for pneumonia were calculated using the number of deaths in a given year as the numerator and the annual population of the same year as the denominator [21]. Age-adjusted mortality rates (AAMRs) were estimated with direct standardization to account for changes in age distribution, with the 2000–2025 WHO population serving as the standard reference [22]. All rates were expressed per 100,000 population, and 95% confidence intervals (CIs) were estimated assuming a Poisson distribution.

Joinpoint regression analysis was used to identify trends in pneumonia mortality rates. The algorithm tests whether a multi-segmented line fits the data better than a straight or less-segmented line, with segments joined at points called joinpoints. The model selection utilized a permutation test, incorporating 4499 permutations, with a maximum of two joinpoints allowed for the entire study period (2006–2019). Confidence intervals were estimated using the empirical quantile method [23].

In addition to best-fit line segments, expressed as annual percentage change (APC), the average APC (AAPC) was calculated for four pre-specified fixed intervals: pre-PCV10 period (2006–2011), early post-PCV10 period (2013–2016), late post-PCV10 period (2017–2019), and entire post-PCV10 period (2013—2019). The year 2012 was considered a transition period and excluded from the AAPC analysis due to the switch from PCV7 to PCV10 in 2012 (Table S2). The late post-PCV10 period was defined as the period after establishing a mature PCV10 program, where disease and colonization by vaccine serotypes contained in the PCV are largely controlled. This typically occurs after at least 4 years of PCV use, with uptake exceeding 70%. Statistical significance for APC and AAPC was set at *p* < 0.05. Non-significant increasing or decreasing trends indicate a stationary trend [23].

Statistical analyses were performed using Python v3.8.8, while joinpoint regression was conducted using Join Point version 5.3.0 (Statistical Research and Applications Branch, National Cancer Institute) [23].

## Results

### Descriptive analysis of annual number of pneumonia-related deaths

From 2006 to 2019, a total of 102,082 deaths due to pneumonia were reported as the underlying cause of death across all age groups in Colombia. The annual number of deaths ranged from 6,465 in 2016 to 9,001 in 2019, peaking in 2017 before slightly decreasing. Throughout this period, adults aged ≥ 60 years consistently had the highest number of deaths compared to other age groups (Table 1). Most deaths (87.7%) were classified as pneumonia or bronchopneumonia with organism unspecified (J18), while unspecified bacterial pneumonia (J15) accounted for 12.2%. Pneumococcal pneumonia (J13) represented less than 0.1% of the total deaths reported (Table S1).

**Table 1:** Annual number of pneumonia-related deaths by age group in Colombia, 2006–2019

Year	Total deaths	Age group					
		< 1 year	1 to 4 years	5 to 17 years	18 to 49 years	50 to 59 years	≥ 60 years
2006	6465 (100%)	663 (10%)	300 (5%)	186 (3%)	599 (9%)	378 (6%)	4339 (67%)
2007	6137 (100%)	612 (10%)	246 (4%)	163 (3%)	588 (10%)	329 (5%)	4199 (68%)
2008	5651 (100%)	570 (10%)	174 (3%)	119 (2%)	570 (10%)	365 (6%)	3853 (68%)
2009	6010 (100%)	470 (8%)	196 (3%)	120 (2%)	641 (11%)	406 (7%)	4177 (70%)
2010	6400 (100%)	366 (6%)	195 (3%)	146 (2%)	643 (10%)	446 (7%)	4604 (72%)
2011	5927 (100%)	392 (7%)	161 (3%)	104 (2%)	543 (9%)	408 (7%)	4319 (73%)
2012	6381 (100%)	397 (6%)	133 (2%)	120 (2%)	579 (9%)	439 (7%)	4713 (74%)
2013	7010 (100%)	326 (5%)	156 (2%)	110 (2%)	635 (9%)	531 (8%)	5252 (75%)
2014	6852 (100%)	254 (4%)	118 (2%)	99 (1%)	534 (8%)	430 (6%)	5417 (79%)
2015	8447 (100%)	294 (3%)	128 (2%)	101 (1%)	616 (7%)	569 (7%)	6739 (80%)
2016	9023 (100%)	285 (3%)	153 (2%)	104 (1%)	759 (8%)	672 (7%)	7050 (78%)
2017	9143 (100%)	327 (4%)	141 (2%)	100 (1%)	676 (7%)	560 (6%)	7339 (80%)
2018	9635 (100%)	318 (3%)	161 (2%)	107 (1%)	741 (8%)	645 (7%)	7663 (80%)
2019	9001 (100%)	221 (2%)	117 (1%)	112 (1%)	644 (7%)	555 (6%)	7352 (82%)

### Descriptive analysis of annual pneumonia mortality rates

**Fig. 1:**
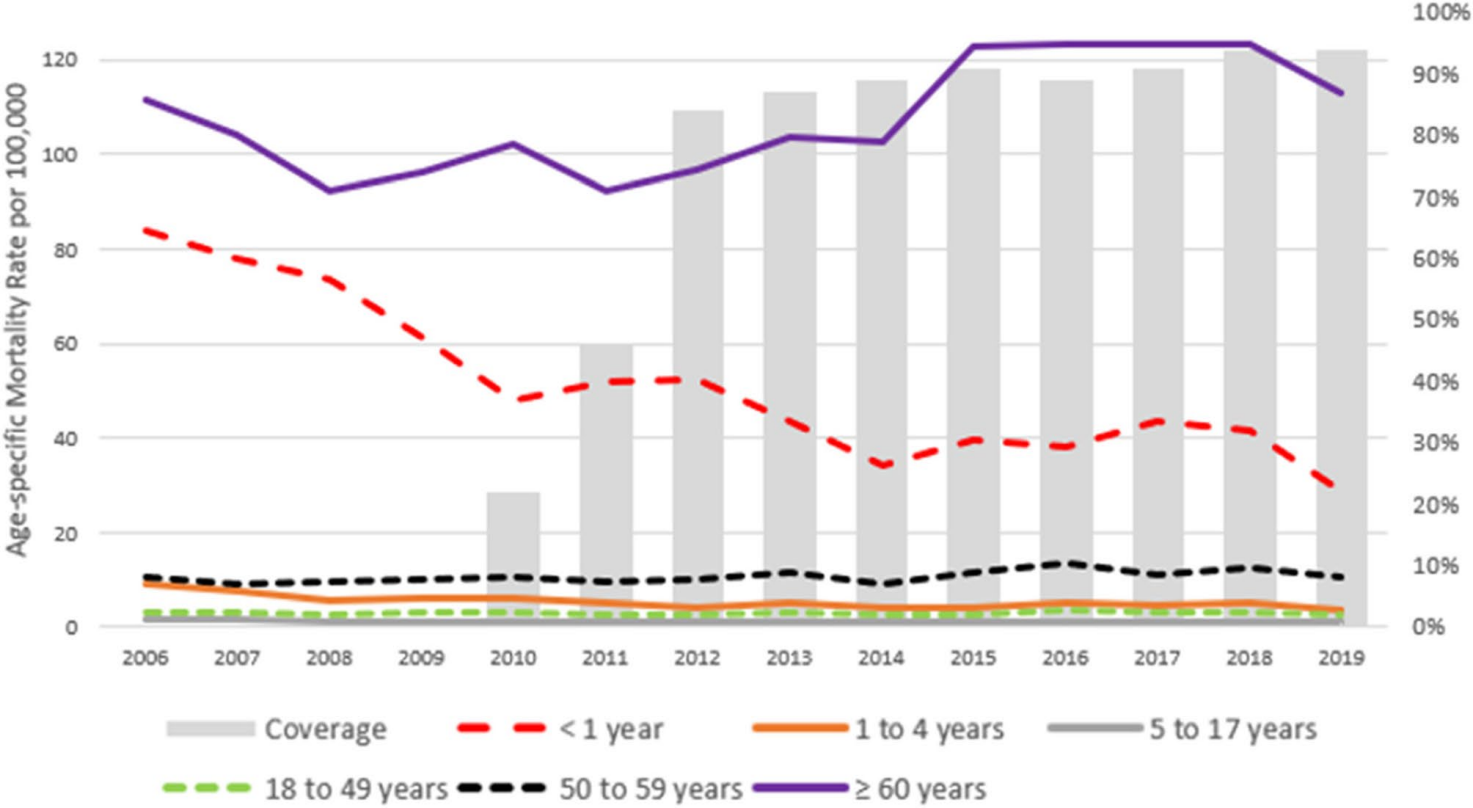
Age group-specific mortality rates of pneumonia per 100,000 population in Colombia, 2006-2019. Infant pneumococcal vaccination coverage (%) is displayed in gray bars. PCV10 was introduced into the national immunization program in 2012 for all infants using a 2+1 schedule at 2, 4, and 12 months of age. According to the official vaccine schedule, these vaccination coverages are considered fully vaccinated children

The annual crude mortality rates and age-adjusted mortality rates throughout the study are described in Table 2. Regarding age-specific mortality rates, the highest rates were observed in adults aged ≥ 60 years, ranging from 111.7 per 100,000 population in 2006 to 112.9 in 2018. Infants < 1 year old had the second highest rates, decreasing from 83.8 deaths per 100,000 population in 2006 to 28.6 in 2019 (Table 2; Fig. 1). Rates in children aged 1–4 years also declined, from 9.3 per 100,000 in 2006 to 3.8 in 2019. For other age groups, mortality rates were relatively low throughout the study period (Table 2; Fig. 1).

**Table 2:** Annual pneumonia mortality rates per 100,000 population according to age group and year of study, Colombian mortality registry, 2011–2019

Year	Crude mortality rate (95% CI)	Age adjusted-mortality rate (95% CI)	Age-specific mortality Rate (95% CI)					
			< 1 year	1 to 4 years	5 to 17 years	18 to 49 years	50 to 59 years	≥ 60 years
2006	15.3 (15.2;15.4)	18.6 (18.1;19.0)	83.8 (83.1;84.5)	9.3 (8.9;9.6)	1.7 (1.5;1.9)	3.0 (2.9;3.2)	10.6 (10.3;10.9)	111.7 (111.4;112)
2007	14.4 (14.3;14.5)	17.1 (16.7;17.6)	78.2 (77.5;78.9)	7.7 (7.0.4;8)	1.5 (1.3;1.7)	2.9 (2.8;3.1)	8.9 (8.6;9.2)	104.2 (103.9;104.5)
2008	13.1 (13.0;13.2)	15.3 (14.9;15.7)	73.6 (72.9;74.3)	5.5 (5.2;5.8)	1.1 (0.9;1.3)	2.8 (2.7;2.9)	9.5 (9.2;9.8)	92.1 (91.8;92.4)
2009	13.8 (13.7;13.9)	15.9 (15.5;16.3)	61.2 (60.5;61.9)	6.3 (5.9;6.6)	1.1 (0.9;1.3)	3.1 (3.0;3.3)	10.2 (9.9;10.5)	96.1 (95.8;96.4)
2010	14.5 (14.4;14.6)	16.5 (16.0;16.9)	48.0 (47.3;48.7)	6.3 (5.9;6.6)	1.4 (1.2;1.5)	3.1 (3.0;3.2)	10.8 (10.5;11.1)	102 (101.7;102.3)
2011	13.3 (13.2;13.4)	14.8 (14.4;15.2)	51.7 (51.0;52.4)	5.2 (4.9;5.6)	1.0 (0.8;1.1)	2.6 (2.4;2.7)	9.5 (9.2;9.8)	92.1 (91.8;92.3)
2012	14.2 (14.1;14.3)	15.4 (15.0;15.8)	52.6 (51.9;53.4)	4.4 (4.0;4.7)	1.1 (0.9;1.3)	2.7 (2.6;2.9)	9.9 (9.6;10.2)	96.7 (96.4;96.9)
2013	15.4 (15.3;15.5)	16.4 (16.0;16.8)	43.4 (42.7;44.1)	5.1 (4.8;5.5)	1.0 (0.8;1.2)	3.0 (2.8;3.1)	11.6 (11.3;11.9)	103.6 (103.4;103.9)
2014	14.9 (14.8;15.0)	15.5 (15.2;15.9)	33.9 (33.2;34.7)	3.9 (3.6;4.3)	0.9 (0.8;1.1)	2.5 (2.3;2.6)	9.1 (8.8;9.4)	102.8 (102.5;103.1)
2015	18.2 (18.1;18.3)	18.5 (18.1;18.9)	39.4 (38.7;40.1)	4.3 (3.9;4.6)	1.0 (0.8;1.1)	2.8 (2.7;2.9)	11.7 (11.5;12.0)	122.9 (122.7;123.2)
2016	19.3 (19.2;19.4)	19.1 (18.7;19.5)	38.2 (37.4;38.9)	5.1 (4.7;5.4)	1.0 (0.8;1.2)	3.4 (3.3;3.6)	13.5 (13.2;13.8)	123.5 (123.3;123.8)
2017	19.3 (19.2;19.4)	18.7 (18.3;19.1)	43.6 (42.9;44.4)	4.7 (4.3;5.0)	1.0 (0.8;1.1)	3.0 (2.9;3.2)	11.0 (10.7;11.3)	123.4 (123.2;123.7)
2018	20.0 (19.9;20.1)	19.0 (18.6;19.3)	41.8 (41.1;42.5)	5.3 (4.9;5.6)	1.0 (0.9;1.2)	3.3 (3.1;3.4)	12.4 (12.1;12.6)	123.3 (123;123.5)
2019	18.2 (18.1;18.3)	16.9 (16.6;17.3)	28.6 (27.9;29.3)	3.8 (3.4;4.1)	1.1 (0.9;1.3)	2.8 (2.6;2.9)	10.4 (10.1;10.7)	112.9 (112.7;113.2)

*CI* Confidence interval. Age-adjusted rate using World Health Organization 2000-2025 as a reference

### Time-series analysis

**Fig. 2:**
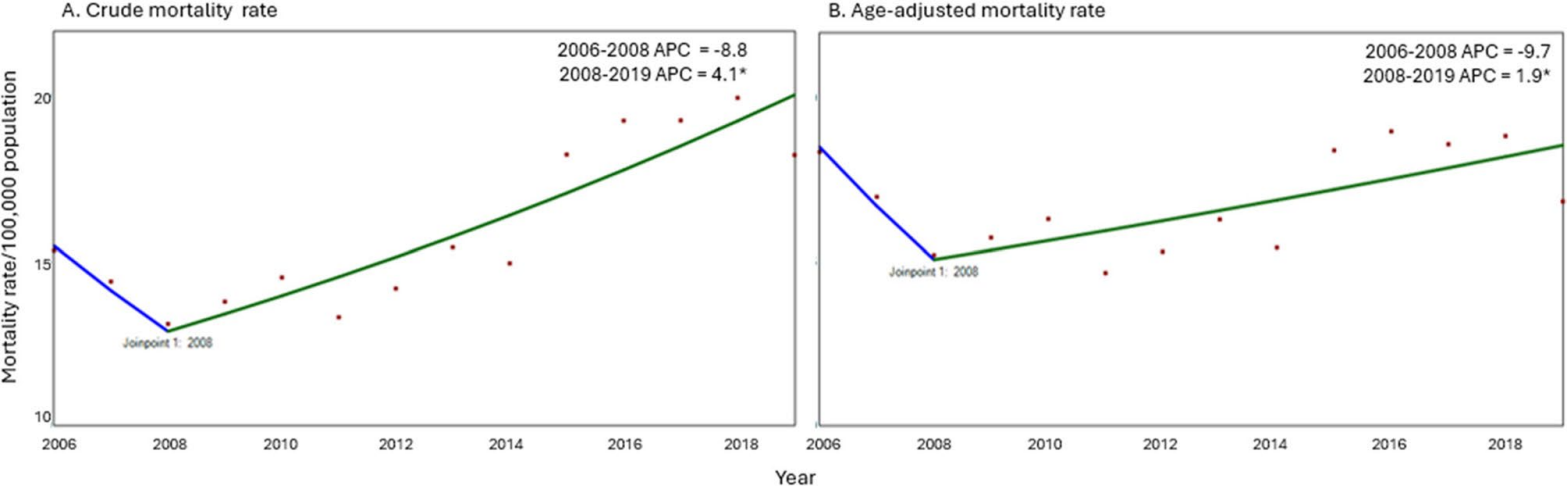
Trends in the pneumonia mortality rates per 100,000 population using joinpoint regression analysis, Colombia, 2006-2019. **A** Crude pneumonia mortality rate. **B** Age-adjusted pneumonia mortality rate. APC, annual percentage change. *Significant trend *p*-value < 0.05. Non-significant increasing or decreasing trends indicate stable trends

**Fig. 3:**
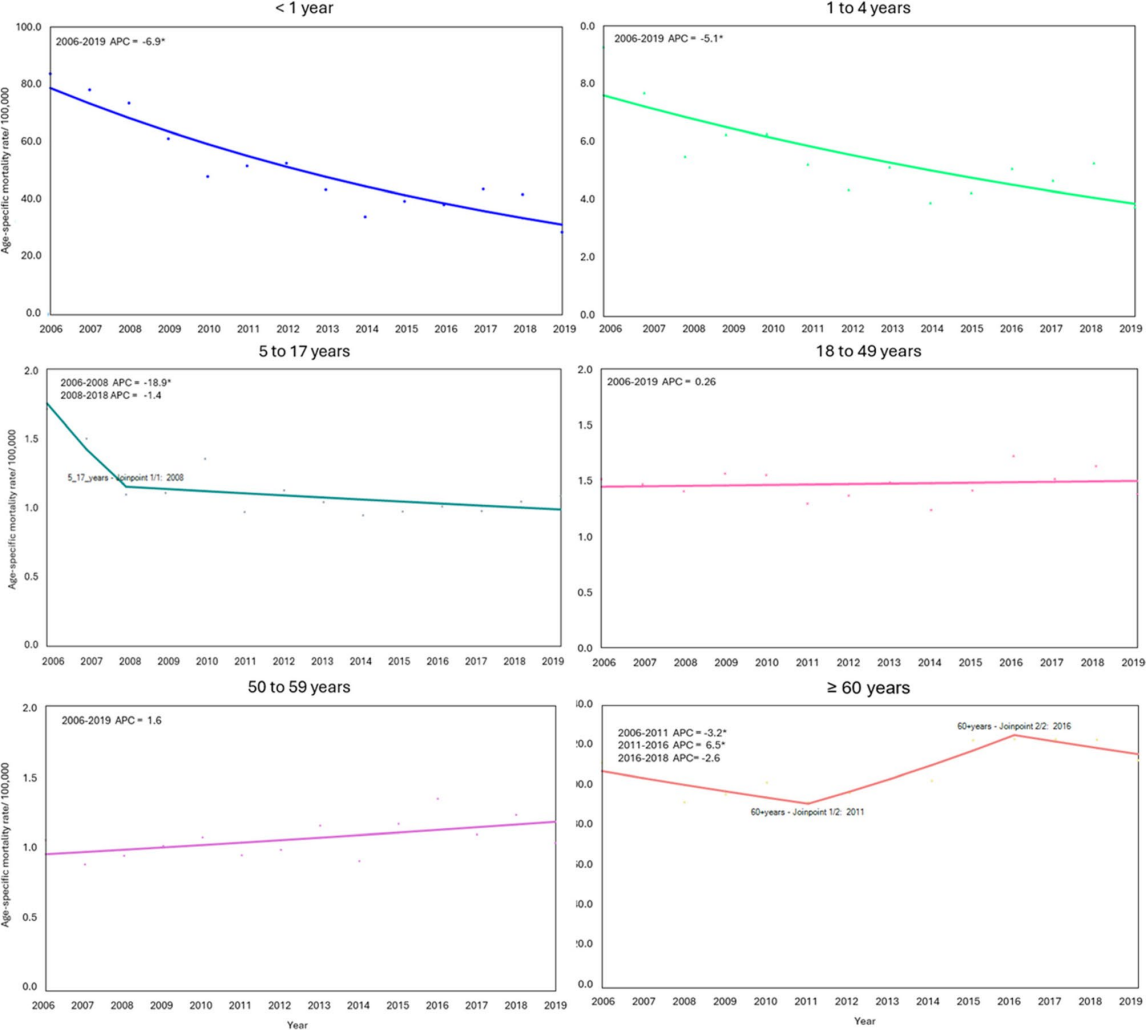
Trends in the pneumonia age-specific mortality rates per 100,000 population using joinpoint regression analysis, Colombia, 2006-2019. APC, annual percentage change. *Significant trend *p*-value < 0.05. Non-significant increasing or decreasing trends indicate stable trends

Using joinpoint regression to analyze time trend inflection (APC) from 2006 to 2019, both the crude and age-adjusted pneumonia mortality rates revealed a significant joinpoint in 2008, dividing the study period into two segments. From 2006 to 2008, both rates showed a stable trend, followed by an increasing trend from 2008 to 2019. The age-adjusted mortality rates increased slightly by 1.9% annually, while the crude mortality rate increased by 4.1% per year (Table 3; Fig. 2). For infants < 1 year and children 1–4 years, the age-specific mortality rates presented a decreasing trend over the study period, with APCs of 6.9% and 5.1%, respectively. For adults aged 18–49 years and 50–59 years, the rates remained stable. Adults aged ≥ 60 years experienced a decreasing trend with an APC of 3.2% per year from 2006 to 2011, followed by an increasing trend of 6.5% per year from 2011 to 2016, and then the mortality rates stabilized from 2016 to 2019 (Table 3; Fig. 3). The AAPC showing pre-PCV10 and post-PCV10 periods, including early and late post-PCV10 analyses can be seen in Table 3.

**Table 3:** Trends in the age-adjusted mortality rate and age-specific mortality rate of pneumonia according to the study period and age group using joinpoint regression analysis. Colombia, 2006–2019

Age Group (years)	Annual percentage change		Average annual percentage change			
	Year	Per time trend inflection	Pre-PCV10 period (2006–2011)	Early post-PCV10 period (2013–2016)	Late post-PCV10 period (2017–2019)	Post-PCV10 period (2013–2019)
CMR	2006–2008	−8.8 (−14.8; 2.4)	−1.2 (−3.7; 2.4)	4.1* (2.9; 6.3)	4.1* (2.9; 7.9)	4.1* (2.9; 6.3)
	2008–2019	4.1* (2.9; 7.9)				
AAMR	2006–2008	−9.7 (−15.4; 0.4)	−2.9 (−5.2; 0.5)	1.9*(0.7;3.9)	1.9*(0.8;5.7)	1.9*(0.8;3.9)
	2008–2019	1.9* (0.7; 5.7)				
< 1 year	2006–2019	−6.9*(−9.3; −4.9)	−6.9*(−9.3; −4.9)	−6.9*(−9.3; −4.9)	−6.9*(−9.3; −4.9)	−6.9*(−9.3; −4.9)
1 to 4 years	2006–2019	−5.1*(−8.0; −2.6)	−5.1*(−8.0; −2.6)	−5.1*(−8.0; −2.6)	−5.1*(−8.0; −2.6)	−5.1*(−8.0; −2.6)
5 to 17 years	2006–2008	−19.0*(−26.9; −4.6)	−8.8* (−12.4; −4.3)	−1.4 (−3.9; 2.3)	−1.4 (−3.3; 6.2)	−1.4 (−3.3; 2.9)
	2008–2019	−1.4 (−3.3; 6.2)				
18 to 49 years	2006–2019	0.3 (−1.2; −1.8)	0.3 (−1.2;1.8)	0.3 (−1.2;1.8)	0.3 (−1.2;1.8)	0.3 (−1.2;1.8)
50 to 59 years	2006–2019	1.6 (−0.2;3.8)	1.6 (−0.2;3.8)	1.6 (−0.2;3.8)	1.6 (−0.2;3.8)	1.6 (−0.2;3.8)
60 years	2006–2011	−3.2*(−9.7; −0.2)	−3.2* (−6.8; −0.2)	6.5* (3.1;11.4)	−2.6 (−9.2; 0.9)	1.9* (0.1; 3.8)
	2011–2016	6.5* (4.0; 12.4)				
	2016–2019	−2.6 (−9.2; 0.9)				

*CMR* Crude mortality rate, *AAMR* Age-adjusted mortality rate, *ASMR* Age-specific mortality rate

*Significant trend *p*-value < 0.05. Non-significant increasing or decreasing trends indicate stable trends

## Discussion

This 14-year time-series analysis, encompassing both pre-PCV10 and post-PCV10 data, provides valuable insights into the effect of pediatric pneumococcal vaccination on pneumonia mortality trends across various age groups in Colombia. Our study revealed significant reductions in age-specific pneumonia mortality rates among children under 5 years throughout the study period. Conversely, no reduction in pneumonia mortality rates was observed in unvaccinated age groups older than 5 years following the introduction of PCV10.

For infants aged < 1 year and children aged 1 to 4 years, the decreasing trend in age-specific mortality rate trends began prior to the PCV10 introduction, suggesting that factors such as the earlier implementation of PCV7 for high-risk infants and better nutrition in Colombia may have played a significant role [2, 3, 24, 25] The developing immune systems of children make them particularly vulnerable to nutritional deficiencies, which can severely impact their overall health [3]. According to the Global Burden of Disease (GBD) 2016 report, childhood wasting is the leading risk factor for mortality from lower respiratory infections in children under five, accounting for 61.4% of such deaths [3]. These findings align with previous studies in Latin America, demonstrating the effect of socioeconomic improvements and introduction of PCV in preventing pneumonia-related mortality in young children [7, 25–27].

Unlike studies reporting reduction in pneumonia mortality in unvaccinated groups post-PCV introduction, our study did not observe this effect in individuals older than 5 years [12, 28]. These findings align with another study carried out in South American countries [17]. Interpretating our study findings in the context of serotyping data is crucial. In 2022, Colombia switched to PCV13 based on local surveillance data indicating that most pneumococcal disease cases were caused by serotypes not covered by PCV10, specifically serotypes 19 A, 3, and 6 C [18, 29]. This serotype replacement affected herd protection, as demonstrated by the increased prevalence of PCV13non PCV10 serotypes in unvaccinated older age groups in Colombia [8, 30]. Consequently, the observed effects in our study might not fully represent the broader potential for herd protection. It is essential to continue evaluating circulating serotypes, ensuring that vaccines are updated to cover the most prevalent and pathogenic serotypes. The introduction of new vaccines, such as PCV15, PCV20 and PCV21, which cover additional serotypes, might provide a more comprehensive understanding of herd protection in the future.

Our findings align with previous studies from the GBD, which reported a stable pattern of pneumonia mortality among older adults [2, 3]. This consistency highlights the often overlooked and increasing burden of pneumonia among adults aged ≥ 60 years, who accounted for more than three-quarters of pneumonia-related deaths in our study in 2019. In Colombia, older adults are defined as those aged ≥ 60 years, with this age threshold integrated into various health programs, including the vaccination schedule for older adults under the NIP, which currently offers seasonal influenza vaccine [20, 31]. Furthermore, the public health policies on Aging and Old Age for 2022–2031, supported by the World Bank, specifically target this age group [31]. Consequently, analyzing data for adults ≥ 60 years provides valuable insights for national programs, aligned with local definitions and public health policies for older adults.

Estimating the population-level effect of PCVs is challenging, particularly in low- and middle-income countries like Colombia, due to diagnostic limitations. As a result, evaluations often focus on nonspecific outcomes like pneumonia rather than pneumococcal pneumonia. In our study, only a small fraction (0.05%) of pneumonia cases were reported as J13 (pneumococcal pneumonia), making it impractical for analysis. To address this, we adopted a standard approach used by other investigators, which includes a broader range of ICD-10 codes that do not specify the causative agent but may still involve *S. pneumoniae* (J15-J18) [7, 17]. This approach provides a unique opportunity to understand the local pneumonia trends and assess the direct and indirect impact of PCV on pneumonia mortality.

This study has several limitations. Our evaluation specifically focused on pneumonia mortality, meaning other infectious etiologies, changes in comorbidity prevalence, or public interventions beyond the pediatric PCV in the NIP likely contributed to the reported pneumonia burden. Additionally, the lack of specific variables may hinder our ability to fully assess the impact of PCV. Although we aimed to emphasize community-acquired pneumonia, where *S pneumoniae* is estimated to be responsible for 38-50% of deaths across various age groups, this detailed information on community versus hospital-acquired infection was not available in the database [3, 32]. To avoid including deaths from hospital-acquired pneumonia, we adopted a methodology similar to that of the GBD 2019, considering only the underlying cause of death for pneumonia [33]. However, this approach may inadvertently include deaths related to hospital-acquired pneumonia. This limitation underscores the need for improved data collection and classification methods to accurately capture the epidemiology of pneumonia.

The strengths of our study include the use of Colombia´s national death registration data, which is categorized as high quality according to WHO standards [34]. The completeness and quality of cause-of-death assignment were maintained by Colombia’s National Administrative Department of Statistics (DANE), which follows stringent quality standards and conducts regular reviews and improvement plans [35, 36]. Furthermore, we analyzed data spanning a 14-year period including data, encompassing both pre-and post-PCV10 periods. The use of joinpoint regression and a permutation test involving 4,499 permutations further strengthened the reliability of our findings by rigorously testing their stability [23].

Our study findings underscore the importance of extending successful interventions, such as vaccination programs to older adults. This is particularly crucial in countries like Colombia, which are experiencing epidemiological transitions with aging populations and increased life expectancy. The data on pneumonia mortality trends across all age groups can inform national immunization policies and guide investments in interventions aimed at protecting older adults. Improving access to primary health services, including mass vaccination and preventive measures, and addressing unfavorable socioeconomic conditions are essential for controlling pneumonia among both children and older adults.

## Conclusions

In the post-PCV10 period (2013–2019), a significant reduction in pneumonia-related mortality was seen in infants < 1 year and children aged 1–4 years. Other age groups exhibited stable trends in post-PCV10 period with no evidence of indirect benefits seen in unvaccinated age groups older than 5 years. Notably, adults ≥ 60 years experienced a higher number of pneumonia-related deaths compared to other age groups, and their age-specific mortality rates remained around 112 cases per 100,000 population throughout this period. The lack of a universal pneumococcal vaccination program for older adults highlights a critical gap in public health measures. To mitigate the pneumonia burden in this vulnerable population, it is imperative to implement additional preventive strategies. Continuous monitoring of pneumococcal disease trends and serotype distribution is essential to ensure that immunization policies remain effective and adaptable to new vaccines, ultimately aiming to reduce pneumonia mortality among both young children and older adults.

## Supplementary Information

Additional file 1.

Additional file 2.

## Data Availability

All data used in this study are publicly available from official Colombian government sources. Mortality data were obtained from the National Department of Statistics (DANE): https://www.dane.gov.co, and health service data were retrieved from the Social Protection Information System (SISPRO): https://www.dane.gov.co.
