# Supplementary material for: Assessing the impact of childhood pneumococcal vaccination on pneumonia mortality in Colombia: a 14-year analysis
Source: BMC Public Health. 2025 Sep 1;25:2990. doi: 10.1186/s12889-025-23631-1 (PMC12400714; doi:10.1186/s12889-025-23631-1)
Supplement: Supplementary file 1 — Additional file 1. [file 12889_2025_23631_MOESM1_ESM.docx]

**Table S1**. Supplementary Table 1. Definition of pneumonia deaths using the International Classification of Diseases (Tenth Edition), Colombia, 2006-2019.

| **Diagnosis** | **ICD-10 codes** | **General mortality database**  **2006-2019** | |  |
| --- | --- | --- | --- | --- |
|  |  |  |  |  |
|  |  | **Frequency** | **%** |  |
| Pneumococcal pneumonia | J13 | 49 | 0.05 |  |
| Bacterial pneumonia, unspecified | J15, J15.0-9 | 12,466 | 12.21 |  |
| Pneumonia due to other infectious organisms, not elsewhere classified | J16, J16.0, J16.8 | 71 | 0.07 |  |
| Pneumonia in diseases classified elsewhere | J17, J17.0-3, J17.8 | 0 | 0.00 |  |
| Pneumonia or bronchopneumonia, organism unspecified | J18.0, J18.1, J18.9 | 89,496 | 87.67 |  |
| Total | | 102,082 | 100 |  |
