# Supplementary material for: Assessing the impact of childhood pneumococcal vaccination on pneumonia mortality in Colombia: a 14-year analysis
Source: BMC Public Health. 2025 Sep 1;25:2990. doi: 10.1186/s12889-025-23631-1 (PMC12400714; doi:10.1186/s12889-025-23631-1)
Supplement: Supplementary file 2 — Additional file 2. [file 12889_2025_23631_MOESM2_ESM.docx]

Table S2. Supplementary Table 2. National pneumococcal vaccination coverage rate for infants, Colombia, 2006-2019.

| Calendar year | PCV Vaccination Coverage (%) |
| --- | --- |
| 2006 | PCV7, only high-risk population |
| 2007 | PCV7, only high-risk population |
| 2008 | PCV7, only high-risk population |
| 2009 | PCV7, only high-risk population |
| 2010 | PCV7- 22 % |
| 2011 | PCV7- 46 |
| 2012 | PCV10, 84 |
| 2013 | PCV10, 87 |
| 2014 | PCV10, 89 |
| 2015 | PCV10, 91 |
| 2016 | PCV10, 89 |
| 2017 | PCV10, 91 |
| 2018 | PCV10, 94 |
| 2019 | PCV10, 94 |

PCV10: 10-valent pneumococcal conjugate vaccine. Note: PCV7 was introduced into the national immunization program in 2006, exclusively targeting infants at high risk or those with certain medical conditions. By 2012, the program expanded to include all infants and transitioned to PCV10, administered using a 2+1 schedule at 2, 4, and 12 months of age. According to the official vaccine schedule, these vaccination coverages are considered fully vaccinated children. **Source:** World Health Organization. Pneumococcal vaccination coverage [cited 2024 Sep 07]. Available from: https://immunizationdata.who.int/global.
